# Supplementary material for: Practical continuous-variable quantum key distribution with composable security
Source: Nat Commun. 2022 Aug 12;13:4740. doi: 10.1038/s41467-022-32161-y (PMC9374721; doi:10.1038/s41467-022-32161-y)
Supplement: Supplementary file 1 — Supplementary Information [file 41467_2022_32161_MOESM1_ESM.pdf]

Practical continuous-variable quantum key distribution with composable security: supplemental document

Nitin Jain,[1,*] Hou-Man Chin,[2,1] Hossein Mani,[1] Cosmo Lupo,[3,4] Dino Solar Nikolic,[1] Arne Kordts,[1] Stefano Pirandola,[5] Thomas Brochmann Pedersen,[6] Matthias Kolb,[7] Bernhard Ömer,[7] Christoph Pacher,[7] Tobias Gehring,[1,†] and Ulrik L. Andersen[1,‡]

[1] *Center for Macroscopic Quantum States (bigQ), Department of Physics, Technical University of Denmark, 2800 Kongens Lyngby, Denmark*
[2] *Department of Photonics, Technical University of Denmark, 2800 Kongens Lyngby, Denmark*
[3] *Department of Physics and Astronomy, University of Sheffield, S3 7RH Sheffield, UK*
[4] *Dipartimento Interateneo di Fisica, Politecnico di Bari, 70126, Bari, Italy*
[5] *Department of Computer Science, University of York, York YO10 5GH, UK*
[6] *Cryptomathic A/S, Aaboulevarden 22, 8000 Aarhus, Denmark*
[7] *Center for Digital Safety & Security, AIT Austrian Institute of Technology GmbH, 1210 Vienna, Austria.*

In the sections below, we detail several aspects of the theoretical security analysis and the experimental system that could not be explained in the main manuscript.

SUPPLEMENTARY NOTE 1: IMPROVED CONFIDENCE INTERVALS

The goal of the parameter estimation routine is to obtain, from empirical data, an estimate, with confidence intervals, of the relevant parameters that characterize the CVQKD protocol. For our protocol it is essential to estimate the variance and covariance of the outcome of unbounded variables, e.g., during the calibration process and for the estimation of the secret key fraction. These unbounded variables are either the outputs of heterodyne detection on the receiver's side, or the preparation variables on the transmitter's side. In all we assume $2n$ input (amplitude and phase quadrature) values that yield us n complex symbols. At the transmitter, we denote the j-th symbol by $(X_{2j-1}, X_{2j}) := (q^j_{\rm tx}, p^j_{\rm tx})$, and correspondingly, the receiver obtains the symbol $(Y_{2j-1}, Y_{2j}) := (q^j_{\rm rx}, p^j_{\rm rx})$ through heterodyne measurements. Under the assumption of symmetry of the quadrature variables, we consider the empirical variance and covariance:

$$\hat{x} := \mathrm{Var}(X) := \frac{1}{2n}\|X\|^2 := \frac{1}{2n}\sum_{j=1}^{2n} X_j^2 = \frac{1}{n}\sum_{j=1}^{n} \frac{\left(q^j_{\rm tx}\right)^2 + \left(p^j_{\rm tx}\right)^2}{2}\ , \tag{1}$$

$$\hat{y} := \mathrm{Var}(Y) := \frac{1}{2n}\|Y\|^2 := \frac{1}{2n}\sum_{j=1}^{2n} Y_j^2 = \frac{1}{n}\sum_{j=1}^{n} \frac{\left(q^j_{\rm rx}\right)^2 + \left(p^j_{\rm rx}\right)^2}{2}\ , \tag{2}$$

$$\hat{z} := \mathrm{Cov}(X,Y) := \frac{1}{2n}\langle X, Y\rangle := \frac{1}{2n}\sum_{j=1}^{2n} X_j Y_j = \frac{1}{n}\sum_{j=1}^{n} \left(\frac{q^j_{\rm tx} q^j_{\rm rx} + p^j_{\rm tx} p^j_{\rm rx}}{2}\right)\ . \tag{3}$$

A rigorous calculation of confidence intervals for these variables was performed in Ref. [1]. Here we follow this approach and derive improved bounds. In particular, we modify Lemma 7 to deal with numerical values instead of the exponential bounds of the χ^2-distribution.

Lemma 1 *For two χ^2-distributed random variables with n degrees of freedom, $||X_1||^2$ and $||X_2||^2$ which are projections into orthogonal subspaces of $||X||^2$ with $2n$ degrees of freedom,*

$$Pr\left[2\,||X_1||^2 \geq a'\,||X||^2\right] = \epsilon\,, \tag{4}$$

where $a' = 2(1 - \mathrm{invcdf}_{\mathrm{Beta}(a,b)}(\epsilon))$ and $\mathrm{invcdf}_{\mathrm{Beta}(a,b)}(\epsilon)$ is the inverse of the cumulative distribution function of the beta distribution $\mathrm{Beta}(a,b)$ with $a = b = \frac{n}{2}$.

---

* nitinj@iitbombay.org
† tobias.gehring@fysik.dtu.dk
‡ ulrik.andersen@fysik.dtu.dk

Proof: It can be shown that (see page 174 of Ref. [2], and page 71 of Ref. [3], noting that the χ^2-distribution is a special case of the Gamma-distribution):

$$\frac{||X_1||^2}{||X_1||^2 + ||X_2||^2} \sim \text{Beta}\left(\frac{n}{2}, \frac{n}{2}\right) . \tag{5}$$

So we can write

$$Pr\left[||X_1||^2 \geq \text{invcdf}_{\text{Beta}\left(\frac{n}{2},\frac{n}{2}\right)}(1-\epsilon)||X||^2\right] = \epsilon . \tag{6}$$

For the cumulative distribution function (cdf) of the beta distribution, which is the regularized incomplete beta function $I_x(a,b) = \frac{\text{B}(x;a,b)}{\text{B}(a,b)}$ (the incomplete beta function divided by the complete beta function), the following property holds (see Ref. [4], page 178):

$$\text{I}_x(a,b) = 1 - \text{I}_{1-x}(b,a) . \tag{7}$$

Furthermore, one can show that for $a = b$

$$\text{invcdf}_{\text{Beta}(a,b)}(1-x) = 1 - \text{invcdf}_{\text{Beta}(a,b)}(x) . \tag{8}$$

Supplementary Equation (6), together with Supplementary Equation (8), and putting $a = b = \frac{n}{2}$, gives Lemma 1. □

Doing the same as above for the upper bound, one also gets

$$Pr\left[2\,||X_1||^2 \leq b'||X||^2\right] \leq \epsilon, \tag{9}$$

where $b' = 2\ \text{invcdf}_{\text{Beta}\left(\frac{n}{2},\frac{n}{2}\right)}(\epsilon)$.

Since Lemma 8 of Ref. [1] depends on Lemma 7, the former has to be adapted as well:

Lemma 2 *For two vectors $X \in \mathbb{R}^{4n}$ and $Y \in \mathbb{R}^{4n}$ and $X_1 \in \mathbb{R}^{2n}$, $Y_1 \in \mathbb{R}^{2n}$ projections in a subspace with dimension n, the following holds:*

$$Pr\left[|\langle X_1, Y_1\rangle - \langle X_2, Y_2\rangle| \leq \frac{1}{4}(a'-b')(||X||^2 + ||Y||^2)\right] \geq 1 - 4\epsilon. \tag{10}$$

Proof: Applying Lemma 1 to the vectors $X_1 + Y_1$ and $X_1 - Y_1$, one can write the following inequalities:

$$b'||X+Y||^2 \leq 2||X_1+Y_1||^2 \leq a'||X+Y||^2 \tag{11}$$

$$-a'||X-Y||^2 \leq -2||X_1-Y_1||^2 \leq -b'||X-Y||^2 \tag{12}$$

Note that each of these four inequality holds with probability larger than $1-\epsilon$. We can also write analogous inequalities for $X_2 + Y_2$ and $X_2 - Y_2$. Also, it can be observed that

$$\langle X_i, Y_i\rangle = \frac{1}{4}||X_i + Y_i||^2 + \frac{1}{4}||X_i - Y_i||^2 . \tag{13}$$

Therefore, adding Supplementary Equation (11) to Supplementary Equation (12) and dividing by 8, one gets a modified version of Lemma 8 of Ref. [1] (with $i \in \{1,2\}$):

$$Pr\left[\langle X_i, Y_i\rangle \leq \frac{1}{4}(b'+a')\langle X, Y\rangle + \frac{1}{8}(a'-b')(||X||^2 + ||Y||^2)\right] \geq 1 - 2\epsilon . \tag{14}$$

and

$$Pr\left[\langle X_i, Y_i\rangle \geq \frac{1}{4}(a'+b')\langle X, Y\rangle - \frac{1}{8}(a'-b')(||X||^2 + ||Y||^2)\right] \geq 1 - 2\epsilon . \tag{15}$$

Combining these latter pair of equations one finally gets Lemma 2. □

Now we want to derive the new confidence region for the covariance matrix. Following Ref. [1], we consider a thought-experiment where the transmitter (the receiver) splits their generated (measured) *real-valued* vectors of length $2n$ in two arbitrary vectors X_1 and X_2 (Y_1 and Y_2). (Note that Ref. [1] used a slightly different notation where

the real vector by the transmitter has size $4n$). Using the measurement outcomes of the first part of their quantum states, $||X_1||^2$ and $||Y_1||^2$, we can estimate a confidence region for the quantum state describing the remaining part of her quantum state, and vice-versa.

We are in particular interested in the estimation of the received quadrature variances: The probability of failure of the estimation is given as in Ref. [1], where $\mathbb{E}$ denotes the expectation values,

$$\begin{aligned} p_{\text{bad}}^{||Y||^2} = Pr\{||Y_1||^2 \geq a \ \mathsf{OR}\ ||Y_2||^2 \geq a \\ \mathsf{OR}\ (||Y_1||^2 \leq a \ \mathsf{AND}\ \mathbb{E}||Y_2||^2 \geq b) \\ \mathsf{OR}\ (||Y_2||^2 \leq a \ \mathsf{AND}\ \mathbb{E}||Y_1||^2 \geq b)\} \leq 6\epsilon\,, \end{aligned} \tag{16}$$

where each line's first condition describes the case where the parameter estimation test of the receiver fails, and the second and third lines describe the cases of an incorrect estimation (with parameter estimation test passed). The same applies, with different bounds, for the estimation of the covariance term (see Ref. [1], pages 20 and 24)

$$\begin{aligned} p_{bad}^{\langle X,Y\rangle} = Pr\,\{\,\langle X_1, Y_1\rangle \leq c \ \mathsf{OR}\ \langle X_2, Y_2\rangle \leq c \\ \mathsf{OR}\ (\langle X_1, Y_1\rangle \geq c \ \mathsf{AND}\ \mathbb{E}\langle X_2, Y_2\rangle \leq d) \\ \mathsf{OR}\ (\langle X_2, Y_2\rangle \geq c \ \mathsf{AND}\ \mathbb{E}\langle X_1, Y_1\rangle \leq d)\} \leq 6\epsilon\,. \end{aligned} \tag{17}$$

In Supplementary Equations (16)-(17), there are multiple estimations with ϵ. The probability that one individual estimation fails is given by 6ϵ, therefore we put $\epsilon' = \epsilon/6$. In turn, the probability that either the estimation of $\|X\|^2$ or $\langle X, Y\rangle$ fails is given by 6ϵ.

The bound for a can be estimated using Lemma 1:

$$a = \frac{a'(\epsilon')}{2}||X||^2\,. \tag{18}$$

For b, one gets from Eq (E39) of Ref. [1]:

$$b = a\left(1 + \frac{20}{\epsilon'}e^{-\frac{n}{16}}\right)\,. \tag{19}$$

The factor c can be calculated using Supplementary Equation (15)

$$c = \frac{1}{4}\left[b'(\epsilon') + a'(\epsilon')\right]\langle X, Y\rangle + \frac{1}{8}\left[b'(\epsilon') - a'(\epsilon')\right]\left(||X||^2 + ||Y||^2\right)\,. \tag{20}$$

To compute d, we can use Eq (E49) of Ref. [1],

$$\begin{aligned} Pr\left[(\mathbb{E}\langle X_2, Y_2\rangle \leq d - \delta) \wedge (\langle X_1, Y_1\rangle \geq c)\right] &\leq \frac{d}{\delta}Pr[\langle X_1, Y_1\rangle - \langle X_2, Y_2\rangle \geq c - d] \\ &\leq \frac{c-\delta}{\delta}Pr\left[\langle X_1, Y_1\rangle - \langle X_2, Y_2\rangle \geq \delta\right] \\ &\leq \frac{c}{\delta}Pr\left[\langle X_1, Y_1\rangle - \langle X_2, Y_2\rangle \geq \delta\right] \\ &\leq \frac{9}{4\epsilon'}Pr\left[\langle X_1, Y_1\rangle - \langle X_2, Y_2\rangle \geq \delta\right] = \epsilon'\,, \end{aligned} \tag{21}$$

whereupon $d = c - \delta$ and $\delta \geq \frac{4c\epsilon'}{9}$ was used. The latter is valid for reasonable parameters, see Ref. [1] page 25 below Eq (E56). Rewriting Supplementary Equation (21), one gains

$$Pr\left[\langle X_1, Y_1\rangle - \langle X_2, Y_2\rangle \geq \delta\right] = \frac{4}{9}{\epsilon'}^2\,. \tag{22}$$

With Lemma 2,

$$\delta = \frac{1}{4}\left(a'' - b''\right)\left(||X||^2 + ||Y||^2\right)\,, \tag{23}$$

where a'' and b'' are defined like a' and b', with their argument ϵ' replaced by ${\epsilon'}^2/9$. One then obtains

$$d = c - \delta = \frac{1}{4}(b' + a')\langle X, Y\rangle + \frac{1}{4}\left(\frac{b' - a'}{2} + b'' - a''\right)\left(||X||^2 + ||Y||^2\right)\,. \tag{24}$$

Finally one gains the bounds by dividing by n :

$$y \leq \frac{1}{2n}\, a'(\epsilon') \left(1 + \frac{20}{\epsilon'} e^{\frac{-n}{16}}\right) ||Y||^2\,, \tag{25}$$

and

$$z \geq \frac{d}{n} = \frac{1}{2n}\langle X, Y\rangle - \frac{1}{4n}\left(\frac{a'(\epsilon') - b'(\epsilon')}{2} + a'({\epsilon'}^2/9) - b'({\epsilon'}^2/9)\right)\left(||X||^2 + ||Y||^2\right)\,. \tag{26}$$

Assuming Gaussianity

In the following we derive confidence intervals under the assumption that the random variables are Gaussian. We summarize our findings as

$$\delta_{\mathrm{Var}}(n,\epsilon) := 1 - \frac{1}{2n}\,\mathrm{invcdf}_{\chi^2_{2n}}(\epsilon)\;, \tag{27}$$

$$\delta_{\mathrm{Cov}}(n,\epsilon) := \frac{1}{2}\left(1 - \frac{1}{2n}\mathrm{invcdf}_{\chi^2_{2n}}\left(\frac{\epsilon}{2}\right)\right)\;. \tag{28}$$

Derivation of confidence interval for the variance: If we assume that the random variables q^j_{rx} and p^j_{rx} are Gaussian with variance y, then

$$\frac{1}{y}\sum_{j=1}^{n}\left[\left(q^j_{\mathrm{rx}}\right)^2 + \left(p^j_{\mathrm{rx}}\right)^2\right] \tag{29}$$

is a chi-square variable with $2n$ degrees of freedom. Therefore we can write

$$Pr\left\{\sum_{j=1}^{n}\left(q^j_{\mathrm{rx}}\right)^2 + \left(p^j_{\mathrm{rx}}\right)^2 < 2n(1-\delta_{\mathrm{Var}})y\right\} = \mathrm{cdf}_{\chi^2_{2n}}(2n(1-\delta_{\mathrm{Var}}))\,. \tag{30}$$

That is,

$$Pr\left\{\sum_{j=1}^{n}\left(q^j_{\mathrm{rx}}\right)^2 + \left(p^j_{\mathrm{rx}}\right)^2 < 2n(1-\delta_{\mathrm{Var}})y\right\} = \epsilon\,, \tag{31}$$

for

$$2n(1-\delta_{\mathrm{Var}}) = \mathrm{invcdf}_{\chi^2_{2n}}(\epsilon)\,. \tag{32}$$

In conclusion, putting

$$\delta_{\mathrm{Var}}(n,\epsilon) := 1 - \frac{1}{2n}\,\mathrm{invcdf}_{\chi^2_{2n}}(\epsilon)\,, \tag{33}$$

we obtain

$$Pr\left\{y > \frac{1}{1-\delta_{\mathrm{Var}}(n,\epsilon)}\,\frac{1}{n}\sum_{j=1}^{n}\frac{\left(q^j_{\mathrm{rx}}\right)^2 + \left(p^j_{\mathrm{rx}}\right)^2}{2}\right\} = \epsilon\,. \tag{34}$$

For $\delta_{\mathrm{Var}}(n,\epsilon) \ll 1$ we can use the approximate bound

$$Pr\left\{y > (1+\delta_{\mathrm{Var}}(n,\epsilon))\,\frac{1}{n}\sum_{j=1}^{n}\frac{\left(q^j_{\mathrm{rx}}\right)^2 + \left(p^j_{\mathrm{rx}}\right)^2}{2}\right\} = \epsilon\,. \tag{35}$$

Finally, with our notation this last expression reads

$$Pr\left\{y > (1+\delta_{\mathrm{Var}}(n,\epsilon))\hat{y}\right\} = \epsilon\,. \tag{36}$$

Derivation of confidence interval for the covariance: For the estimation of the covariance, we need to consider the random variable

$$\sum_{j=1}^{n} q^j_{\mathrm{tx}} q^j_{\mathrm{rx}} + p^j_{\mathrm{tx}} p^j_{\mathrm{tx}} = \frac{1}{4}\sum_{j=1}^{n}\left(q^j_{\mathrm{tx}} + q^j_{\mathrm{rx}}\right)^2 + \left(p^j_{\mathrm{tx}} + p^j_{\mathrm{rx}}\right)^2 - \left(q^j_{\mathrm{tx}} - q^j_{\mathrm{rx}}\right)^2 - \left(p^j_{\mathrm{tx}} - p^j_{\mathrm{rx}}\right)^2\,. \tag{37}$$

Note that the variables

$$\frac{1}{4}\sum_{j=1}^{n}\left(q^j_{\mathrm{tx}} + q^j_{\mathrm{rx}}\right)^2 + \left(p^j_{\mathrm{tx}} + p^j_{\mathrm{rx}}\right)^2\,, \tag{38}$$

$$\frac{1}{4}\sum_{j=1}^{n}\left(q^j_{\mathrm{tx}} - q^j_{\mathrm{rx}}\right)^2 + \left(p^j_{\mathrm{tx}} - p^j_{\mathrm{rx}}\right)^2 \tag{39}$$

are statistically independent and are (not normalized) χ^2 variables with $2n$ degrees of freedom.

Applying the results of previous section we have:

$$Pr\left\{\frac{1}{4}\sum_{j=1}^{n}\left(q^j_{\mathrm{tx}} + q^j_{\mathrm{rx}}\right)^2 + \left(p^j_{\mathrm{tx}} + p^j_{\mathrm{rx}}\right)^2 > 2n(1+\delta^+_{\mathrm{Var}})z_+\right\} = 1 - \mathrm{cdf}_{\chi^2_{2n}}(2n(1+\delta^+_{\mathrm{Var}}))\,, \tag{40}$$

$$Pr\left\{\frac{1}{4}\sum_{j=1}^{n}\left(q^j_{\mathrm{tx}} - q^j_{\mathrm{rx}}\right)^2 + \left(p^j_{\mathrm{tx}} - p^j_{\mathrm{rx}}\right)^2 < 2n(1-\delta^-_{\mathrm{Var}})z_-\right\} = \mathrm{cdf}_{\chi^2_{2n}}(2n(1-\delta^-_{\mathrm{Var}}))\,, \tag{41}$$

where we have put

$$z_+ = \mathbb{E}\left[\frac{1}{4}\left(q^j_{\mathrm{tx}} + q^j_{\mathrm{rx}}\right)^2\right] = \mathbb{E}\left[\frac{1}{4}\left(p^j_{\mathrm{tx}} + p^j_{\mathrm{rx}}\right)^2\right]\,, \tag{42}$$

$$z_- = \mathbb{E}\left[\frac{1}{4}\left(q^j_{\mathrm{tx}} - q^j_{\mathrm{rx}}\right)^2\right] = \mathbb{E}\left[\frac{1}{4}\left(p^j_{\mathrm{tx}} - p^j_{\mathrm{rx}}\right)^2\right]\,, \tag{43}$$

$$z = z_+ - z_-\,. \tag{44}$$

For $\delta^{\pm}_{\mathrm{Var}} \ll 1$ we can write the approximate expressions:

$$Pr\left\{z_+ < \frac{1-\delta^+_{\mathrm{Var}}}{8n}\sum_{j=1}^{n}\left(q^j_{\mathrm{tx}} + q^j_{\mathrm{rx}}\right)^2 + \left(p^j_{\mathrm{tx}} + p^j_{\mathrm{rx}}\right)^2\right\} = 1 - \mathrm{cdf}_{\chi^2_{2n}}(2n(1+\delta^+_{\mathrm{Var}}))\,, \tag{45}$$

$$Pr\left\{z_- > \frac{1+\delta^-_{\mathrm{Var}}}{8n}\sum_{j=1}^{n}\left(q^j_{\mathrm{tx}} - q^j_{\mathrm{rx}}\right)^2 + \left(p^j_{\mathrm{tx}} - p^j_{\mathrm{rx}}\right)^2\right\} = \mathrm{cdf}_{\chi^2_{2n}}(2n(1-\delta^-_{\mathrm{Var}}))\,, \tag{46}$$

which in turn imply

$$Pr\left\{z < \frac{1-\delta^+_{\mathrm{Var}}}{8n}\sum_{j=1}^{n}\left(q^j_{\mathrm{tx}} + q^j_{\mathrm{rx}}\right)^2 + \left(p^j_{\mathrm{tx}} + p^j_{\mathrm{rx}}\right)^2 - \frac{1+\delta^-_{\mathrm{Var}}}{8n}\sum_{j=1}^{n}\left(q^j_{\mathrm{tx}} - q^j_{\mathrm{rx}}\right)^2 + \left(p^j_{\mathrm{tx}} - p^j_{\mathrm{rx}}\right)^2\right\}$$
$$\leq 1 - \mathrm{cdf}_{\chi^2_{2n}}(2n(1+\delta^+_{\mathrm{Var}})) + \mathrm{cdf}_{\chi^2_{2n}}(2n(1-\delta^-_{\mathrm{Var}}))\,. \tag{47}$$

The above is equivalent to

$$Pr\left\{z < \hat{z} - \frac{\delta^+_{\mathrm{Var}}}{8n}\sum_{j=1}^{n}\left(q^j_{\mathrm{tx}} + q^j_{\mathrm{rx}}\right)^2 + \left(p^j_{\mathrm{tx}} + p^j_{\mathrm{rx}}\right)^2 - \frac{\delta^-_{\mathrm{Var}}}{8n}\sum_{j=1}^{n}\left(q^j_{\mathrm{tx}} - q^j_{\mathrm{rx}}\right)^2 + \left(p^j_{\mathrm{tx}} - p^j_{\mathrm{rx}}\right)^2\right\}$$
$$\leq 1 - \mathrm{cdf}_{\chi^2_{2n}}(2n(1+\delta^+_{\mathrm{Var}})) + \mathrm{cdf}_{\chi^2_{2n}}(2n(1-\delta^-_{\mathrm{Var}}))\,. \tag{48}$$

Put for simplicity $\delta = \max\{\delta^{-}_{\mathrm{Var}}, \delta^{-}_{\mathrm{Var}}\}$:

$$Pr\left\{ z < \hat{z} - \frac{\delta}{4n} \sum_{j=1}^{n} \left(q^j_{\mathrm{tx}}\right)^2 + \left(q^j_{\mathrm{rx}}\right)^2 + \left(p^j_{\mathrm{tx}}\right)^2 + \left(p^j_{\mathrm{rx}}\right)^2 \right\}$$
$$\leq 1 - \mathrm{cdf}_{\chi^2_{2n}}\left(2n(1+\delta)\right) + \mathrm{cdf}_{\chi^2_{2n}}\left(2n(1-\delta)\right). \tag{49}$$

In our notation, $\hat{x} = \frac{1}{2n}\sum_{j=1}^{n}\left(q^j_{\mathrm{tx}}\right)^2 + \left(p^j_{\mathrm{tx}}\right)^2$, and $\hat{y} = \frac{1}{2n}\sum_{j=1}^{n}\left(q^j_{\mathrm{rx}}\right)^2 + \left(p^j_{\mathrm{rx}}\right)^2$, therefore the last expression reads:

$$Pr\left\{ z < \hat{z} - \frac{\delta}{2}\left(\hat{x} + \hat{y}\right) \right\} \leq 1 - \mathrm{cdf}_{\chi^2_{2n}}\left(2n(1+\delta)\right) + \mathrm{cdf}_{\chi^2_{2n}}\left(2n(1-\delta)\right). \tag{50}$$

We can further approximate

$$Pr\left\{ z < \hat{z} - \frac{\delta}{2}\left(\hat{x} + \hat{y}\right) \right\} \leq 2\,\mathrm{cdf}_{\chi^2_{2n}}\left(2n(1-\delta)\right). \tag{51}$$

Finally, if we put

$$\delta = 1 - \frac{1}{2n}\mathrm{invcdf}_{\chi^2_{2n}}\left(\frac{\epsilon}{2}\right), \tag{52}$$

the latter reads

$$Pr\left\{ z < \hat{z} - \left(1 - \frac{1}{2n}\mathrm{invcdf}_{\chi^2_{2n}}\left(\frac{\epsilon}{2}\right)\right)\frac{\hat{x}+\hat{y}}{2} \right\} \leq \epsilon. \tag{53}$$

In conclusion, to have the result stated in our notation, we need to define:

$$\delta_{\mathrm{Cov}}(n,\epsilon) := \frac{1}{2}\left(1 - \frac{1}{2n}\mathrm{invcdf}_{\chi^2_{2n}}\left(\frac{\epsilon}{2}\right)\right), \tag{54}$$

which yields the desired relation:

$$Pr\left\{ z < \left(1 - \delta_{\mathrm{Cov}}(n,\epsilon)\frac{\hat{x}+\hat{y}}{\hat{z}}\right)\hat{z} \right\} \leq \epsilon. \tag{55}$$

67 **Further optimization of the confidence interval for covariance estimation**

Let us first show the optimization under the assumption of Gaussianity. Note that instead of Supplementary Equation (37), we could have used the following identity

$$\sum_{j=1}^{n} q^j_{\mathrm{tx}}q^j_{\mathrm{rx}} + p^j_{\mathrm{tx}}p^j_{\mathrm{tx}} = \frac{1}{4}\sum_{j=1}^{n}\left(\lambda q^j_{\mathrm{tx}} + \lambda^{-1}q^j_{\mathrm{rx}}\right)^2 + \left(\lambda p^j_{\mathrm{tx}} + \lambda^{-1}p^j_{\mathrm{rx}}\right)^2$$
$$- \left(\lambda q^j_{\mathrm{tx}} - \lambda^{-1}q^j_{\mathrm{rx}}\right)^2 - \left(\lambda p^j_{\mathrm{tx}} - \lambda^{-1}p^j_{\mathrm{rx}}\right)^2, \tag{56}$$

which holds for any $\lambda > 0$. This would then yield, instead of Supplementary Equation (55), the following confidence interval for the covariance

$$Pr\left\{ z < \left(1 - \delta_{\mathrm{Cov}}(n,\epsilon)\frac{\lambda^2\hat{x} + \lambda^{-2}\hat{y}}{\hat{z}}\right)\hat{z} \right\} \leq \epsilon. \tag{57}$$

The optimal value of λ^2 is the one that minimizes $\lambda^2\hat{x} + \lambda^{-2}\hat{y}$, i.e., $\lambda^2 = \sqrt{\hat{y}/\hat{x}}$. This finally yields the optimised confidence interval:

$$Pr\left\{ z < \left(1 - 2\,\delta_{\mathrm{Cov}}(n,\epsilon)\frac{\sqrt{\hat{x}\,\hat{y}}}{\hat{z}}\right)\hat{z} \right\} \leq \epsilon. \tag{58}$$

Name	Description	Value
ϵ_{cal}	Calibration (both transmitter and receiver)	10^{-10}
ϵ_h	Hashing function	10^{-10}
ϵ_s	Smooth min-entropy	10^{-10}
ϵ_{ent}	Empirical estimate entropy	10^{-10}
$\epsilon_{\rm PE}$	Parameter estimation (PE)	10^{-10}
$\epsilon_{\rm IR}$	Information reconciliation (IR)	10^{-12}
$\epsilon_{\rm qrng}$	Quantum random number generation	2×10^{-6}

Supplementary Table I. Various security parameters (epsilons) in the key length expression.

The same argument can be applied also without assuming Gaussianity, and Supplementary Equation (13) can be replaced by the identity

$$\langle X_i, Y_i \rangle = \frac{1}{4}||\lambda X_i + \lambda^{-1} Y_i||^2 + \frac{1}{4}||\lambda X_i - \lambda^{-1} Y_i||^2 \,. \tag{59}$$

In this way, instead of Supplementary Equation (26) we obtain

$$z \geq \frac{1}{2n}\langle X, Y \rangle - \frac{1}{4n}\left(\frac{a'(\epsilon') - b'(\epsilon')}{2} + a'({\epsilon'}^2/9) - b'({\epsilon'}^2/9)\right)\left(\lambda||X||^2 + \lambda^{-1}||Y||^2\right) \,. \tag{60}$$

Finally, this expression can be optimized by putting $\lambda = ||Y||/||X||$. This yields

$$z \geq \frac{1}{2n}\langle X, Y \rangle - \frac{1}{2n}\left(\frac{a'(\epsilon') - b'(\epsilon')}{2} + a'({\epsilon'}^2/9) - b'({\epsilon'}^2/9)\right) ||X||\,||Y|| \,. \tag{61}$$

SUPPLEMENTARY NOTE 2: SECRET KEY CALCULATION

Here we provide details of the various terms in the composable key length calculation [1, 5]. An expression for the lower bound on the composably secure key length is as follows:

$$\begin{aligned} s_n^{\epsilon_{\rm qrng}+\epsilon_h+\epsilon_s+\epsilon_{\rm IR}+\epsilon_{\rm ent}+\epsilon_{\rm PE}+\epsilon_{\rm cal}} \geq\; & n'\left[\hat{H}(\bar{Y})_\rho - I(Y;E)_{\rho_G}\right] - \mathrm{leak}_{\rm IR}(n', \epsilon_{\rm IR}) \\ & - \log\left(n'\right) \cdot \sqrt{2n' \log\left(2/\epsilon_{\rm ent}\right)} - \sqrt{n'}\,\Delta_{\rm AEP}\left(\frac{p}{3}\epsilon_s^2, d_{\rm rx}\right) \\ & + \log\left(p - \frac{p}{3}\epsilon_s^2\right) + 2\log\left(\sqrt{2}\epsilon_h\right), \end{aligned} \tag{62}$$

where n' is the total number of exchanged and corrected quantum symbols, $H(\bar{Y})_\rho$ is an entropy estimator, $I(Y;E)_{\rho_G}$ represents the Holevo information (with ρ_G being a Gaussian state having the same covariance matrix as ρ), p is the success probability of error correction per frame, $d_{\rm rx}$ is the bit resolution at the receiver, and $\Delta_{\rm AEP}$ relates the asymptotic limit / infinite number of channel uses with the practical finite-size regime (AEP : asymptotic equipartition property). The various epsilons are summarized in Supplementary Table I. Note that $\epsilon_{\rm cal}$, $\epsilon_{\rm PE}$, and $\epsilon_{\rm qrng}$ do not appear in the RHS of Supplementary Equation (62) as their role is implicit, for instance, $\epsilon_{\rm cal}$ quantifies the probability that the transmitter or receiver calibration is incorrect. Also $\epsilon_{\rm qrng}$, which represents the overall security parameter of the random numbers at Tx, is limited by the confidence level of the digitization errors in the QRNG [6].

The final security parameter that quantifies the composable security of our CVQKD system is $\epsilon = \epsilon_{\rm qrng} + \epsilon_{\rm sec} + \epsilon_{\rm cor} + \epsilon_{\rm ent} + \epsilon_{\rm PE} + \epsilon_{\rm cal}$ with $\epsilon_{\rm sec} = \epsilon_h + \epsilon_s$ quantifying the secrecy, and $\epsilon_{\rm cor} = \frac{n'\epsilon_{\rm IR}}{L_{\rm IR}}$ the correctness of the protocol. The block length $L_{\rm IR}$ is dependent on the intricacies of the IR procedure, for instance, we use $L_{\rm IR} = 512000$.

Positivity of min-entropy for a separable state

Given a bipartite state ρ_{AB} and with I_A denoting the identity operator over A, the smooth-min entropy is

$$H_{\min}(A|B)_\rho := \max_\sigma \sup\{\lambda \in \mathbb{R} : \rho_{AB} \leq 2^{-\lambda} I_A \otimes \sigma_B\}, \tag{63}$$

with the maximum taken over all sub-normalized states $\sigma_B \in \mathcal{S}_\leq(\mathcal{H}_B)$ [7, Definition 4.1]. Note that

$$H_{\min}(A|B)_\rho \geq \tilde{H}^{\downarrow}_{\infty}(A|B)_\rho := \sup\{\lambda \in \mathbb{R} : \rho_{AB} \leq 2^{-\lambda} I_A \otimes \rho_B\}, \tag{64}$$

where $\tilde{H}^{\downarrow}_{\infty}(A|B)_\rho$ can also be defined as the limiting case for $\alpha = \infty$ of a quantum conditional Rényi entropy $\tilde{H}^{\downarrow}_{\alpha}(A|B)_\rho$ defined in Ref. [8, Eq (5.18)].

For separable ρ_{AB}, we may write (following Ref. [8, Lemma 5.2])

$$\rho_{AB} = \sum_k p_k \theta^k_A \otimes \rho^k_B \leq \sum_k p_k I_A \otimes \rho^k_B = I_A \otimes \rho_B. \tag{65}$$

This leads to $\tilde{H}^{\downarrow}_{\infty}(A|B)_\rho \geq 0$, since we are left to find the *maximum* value $\lambda_{\max}$ of $\lambda \in \mathbb{R}$ such that simultaneously satisfies

$$\rho_{AB} \leq I_A \otimes \rho_B,\ \rho_{AB} \leq 2^{-\lambda} I_A \otimes \rho_B. \tag{66}$$

Clearly it must be $\lambda_{\max} \geq 0$.

SUPPLEMENTARY NOTE 3: FUNCTIONAL BLOCKS

Below we provide details of the various blocks depicted in Fig. 2 of the main paper.

Quantum randomness generation

We employed a quantum random number generator (QRNG) based on homodyne measurements of the vacuum state [9]. As a first step, the QRNG generated uniformly distributed random bit strings by real-time Toeplitz hashing on a field programmable gate array (FPGA). The available min-entropy was bounded by a metrological characterization of the device, compatible with the required security parameter [6]. The uniformly distributed bits were then transformed using the inversion sampling method based on the cumulative distribution function [10] to Gaussian-distributed integers with a resolution of 6 bits covering 7 standard deviations. We considered these integers in pairs to form the 'quantum data symbol' train, i.e., the complex amplitudes $X = X_I + iX_Q$ of the coherent states used for IQ modulation, as explained in the next subsection. Supplementary Figures 1(a) and (b) show the histograms for X_I and X_Q, respectively, using a total of 10^9 quantum data symbols.

Modulation (quantum data & pilot tone)

Assuming each quantum data symbol occupying 10 ns in time (quantum data bandwidth $B = 100\,\text{MHz}$), we created two baseband waveforms $X_I(t)$ and $X_Q(t)$ by upsampling the quantum symbol train to 1 GSps with interpolation performed using a root-raised-cosine (RRC) filter, characterized by a rolloff factor of 0.2 and span of 20 symbols. Note that the variable t is the discretized/sampling time instant with a tick of 1 ns. Supplementary Figures 1(c) and (d) show the first 100 symbols and the corresponding 1μs-wide snapshot of the RRC interpolated waveforms.

For sideband modulation and providing a phase reference to the receiver, we upconverted baseband waveforms to a frequency $\Omega_u/2\pi = 200\,\text{MHz}$ and multiplexed a pilot tone in frequency at $\Omega_p/2\pi = 25\,\text{MHz}$, respectively, as depicted in the inset of Fig. 2 of the main paper. The resulting pair of RF waveforms in the time domain are given by

$$RF1(t) = X_I(t)\cos(\Omega_u t) - X_Q(t)\sin(\Omega_u t) + A_p\cos(\Omega_p t),$$
$$RF2(t) = X_I(t)\sin(\Omega_u t) + X_Q(t)\cos(\Omega_u t) + A_p\sin(\Omega_p t).$$

with A_p being a variable for controlling the power in the pilot tone w.r.t. the quantum data band.

Automatic bias control

In our experiment, we implement the optical single sideband (OSSB) encoding scheme using an IQ modulator, which consists of two nested Mach-Zehnder modulators (MZMs) and a phase modulator (PM). Each of these sub

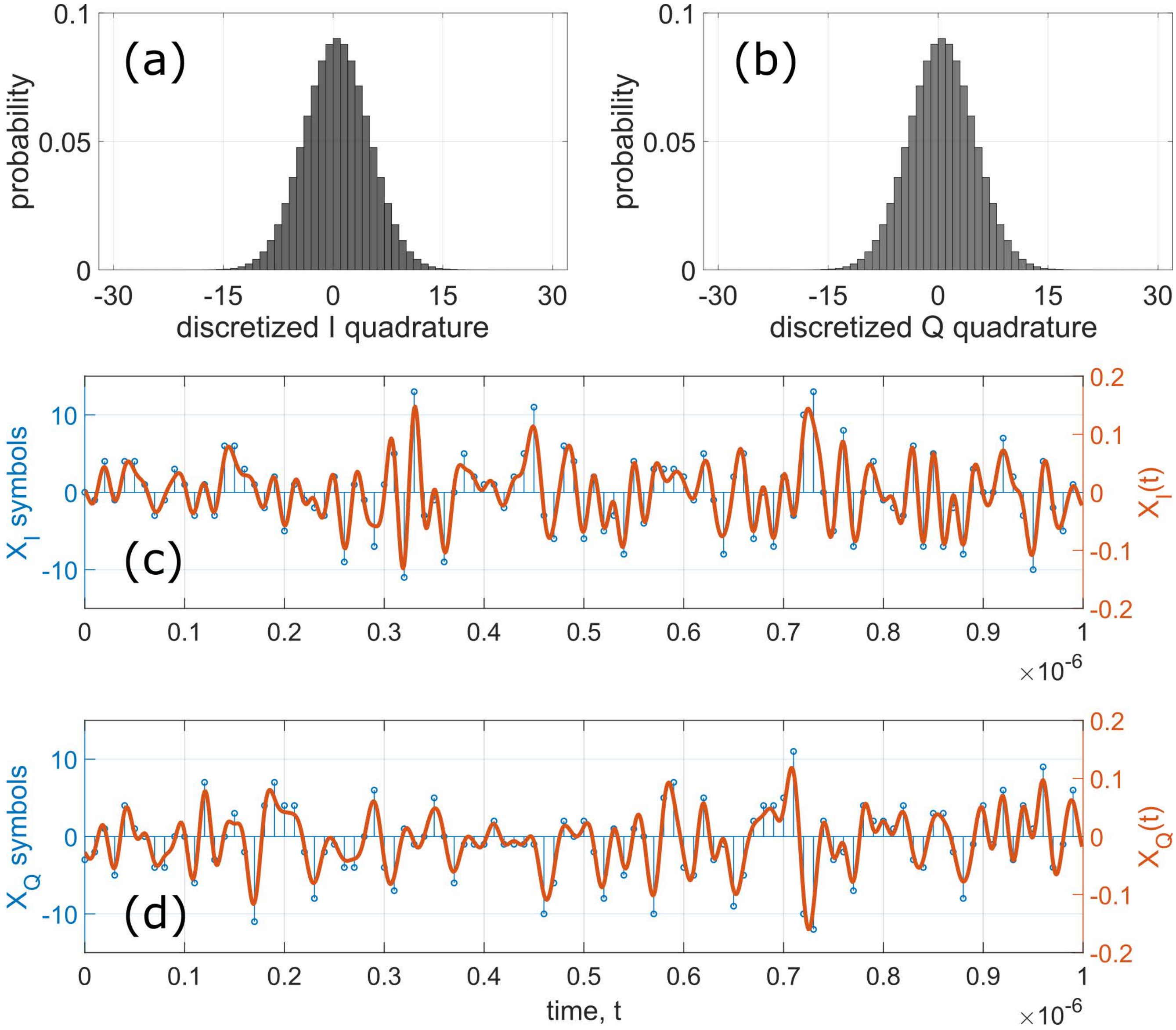

Supplementary Figure 1. From raw I and Q symbols to digital waveforms. (a,b): Histograms of the uniform-to-Gaussian converted symbols X_I, X_Q. (c,d) The process of creating the digital waveforms involved upsampling (from the symbol bandwidth of 100 MHz to sampling frequency of 1 GHz) while interpolating with a root-raised-cosine filter having a span of 20 symbols.

modulators is characterized by a voltage V_π. For the PM, the incoming light wave experiences a π phase shift at the output, if the applied DC bias voltage is $V_{DC}^{\text{PM}} = V_\pi$. The optical transmission through the MZM becomes maximum [minimum] on applying $V_{DC}^{\text{MZM}} = kV_\pi$ if k is an even [odd] integer.

Due to environmental drifts etc., the parameter V_π however exhibits fluctuations, and may require active control. An automatic bias controller continually updates the DC bias voltages by tracking such variations (using a photodiode and a logic circuit for feedback). With appropriate DC biases along with the RF waveforms described in the previous subsection, the optical output shows carrier and sideband suppression, which enables OSSB encoding.

Heterodyne detection, acquisition and demodulation

The technique of RF heterodyne detection enables simultaneous measurement of both I and Q quadrature components of the received signal. Heterodyne detection requires that the signal and the local oscillator (LO) have distinct center frequencies. In our experiment, we had a frequency detuning of around 320 MHz, i.e., the signal and LO gave

rise to an interference or 'beat' signal at the output of the detector; see the right inset of Fig. 2 in the main paper.

We inserted a low pass filter (cutoff $\approx$ 365 MHz) at the detector output for reducing high frequency noise. The filtered output was captured at a sampling rate of 1.0 GSps by an analog-to-digital converter (ADC) inside a fast acquisition card, externally triggered by the AWG. We also used an external 10 MHz clock reference to synchronize the timebase of the AWG with that of the acquisition card. The acquired dataset is divided into 'frames', each of length 10^7 samples.

The process of reconstructing the transmitted symbols at the receiver required extensive digital signal processing (DSP), which was performed offline on a frame-to-frame basis. In particular, the DSP used a machine learning based approach for optimally tracking the phase between the Tx and Rx lasers (which are free running in our system). The machine learning framework is based on an unscented Kalman filter (UKF), and the primary advantage of doing carrier phase recovery via this approach is the ability to make use of fairly low power pilot tones—in fact, the power of the pilot tone in our experiment is of the same order as the power contained in the quantum signal band [11]. Note that at such signal-to-noise ratios, standard phase recovery methods become quite inaccurate. Below we detail some of the DSP routines done on the acquired data frames:

1. **Pilot frequency estimation:** The pilot frequency was estimated by widely bandpass filtering around the *desired* pilot tone frequency as extracted from a coarsely resolved power spectrum; see the inset on the right in Fig. 2 of the main manuscript. A Hilbert transform was applied to the filtered pilot allowing for extraction of the phase profile by taking its argument. A linear fit yields an estimate of the frequency offset. The procedure was then repeated with a narrower bandpass filter.

2. **Phase estimation:** Using the obtained pilot frequency estimate, we shifted the pilot signal to baseband and downsampled it from the ADC sampling rate of 1.0 GHz to the symbol rate of 100 MHz. We use this signal as the input to the UKF.

3. **Down conversion and phase compensation:** We shifted the quantum data signal also to baseband using the pilot frequency estimate and the known frequency offset between quantum signal and pilot tone ($200-25=175\,\text{MHz}$; see Fig. 2 in the main paper) at the Tx. The phase of the pilot tone obtained from the previous stage was used for correcting the phase of the quantum signal.

4. **Timing recovery** By means of cross correlation between the (upsampled version of) reference / Tx symbols from the appropriate frame, and the down-converted quantum signal from the previous stage, we obtained the temporal shift (due to channel propagation and various electronic delays) between the Tx and Rx. The complex quantum signal after synchronization in both quadratures then yields waveforms $Y_I(t)$ and $Y_Q(t)$ corresponding to that frame.

5. **Filtered downsampling:** We used the same RRC filter as explained in subsection to downsample the quantum signal and obtain the final Rx symbols $Y = Y_I + Y_Q$ corresponding to the frame.

SUPPLEMENTARY NOTE 4: EVALUATION OF TRUSTED PARAMETERS

In an ideal world with perfect components, the signal after the quantum channel would not suffer any further loss or incur more noise. As this is not true in practice, the loss and noise owing to the imperfect components that make up the receiver can either be *untrusted*, i.e., fully attributed to Eve, or *trusted* under the assumption that the receiver is physically inaccessible for Eve. We operate in the latter regime, which requires us then to carefully estimate the trusted parameters and incorporate them in the security proof.

With reference to Fig. 2 from the main paper, the optical loss due to the PC, the (extra, i.e., above 3 dB) loss from the 50/50 BS, and the overall quantum efficiency of the balanced detector contribute to the trusted loss of the receiver. Combining them, we estimated a trusted efficiency $\tau = 0.69$, translating to a loss of 1.6 dB.

To obtain the trusted noise t, we first acquired 10^{10} ADC samples with the Rx laser on and the Tx laser off. We calculated the (inverse of the) averaged frequency response from the acquired data samples to create a 'whitening' filter. Applying such a filter on the original dataset itself yielded a flat response across the entire spectrum, as is illustrated in Supplementary Figure 2(left) by the dotted-blue trace. Note we excluded from this whitening process a small low-frequency region (DC to $\lesssim 1\,\text{MHz}$) and a high-frequency region ($400-500\,\text{MHz}$), depicted by the grey rectangles in Supplementary Figure 2(left), where no components-of-interest are expected. We obtained the dashed-green trace on applying the whitening filter to data samples acquired with both Tx and Rx lasers off. The whitened data samples from both these measurements were subjected to the demodulation procedure outlined in subsection , using the information about the frequency band occupied by the quantum data.

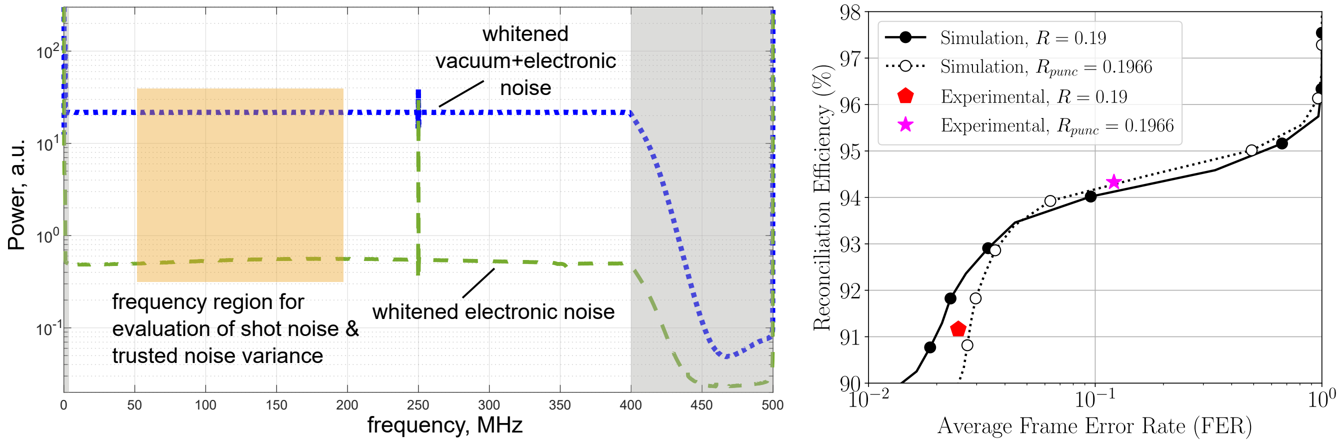

Supplementary Figure 2. (Left) Receiver calibration for shot noise and trusted noise variances. (Right) Influence of puncturing on information reconciliation efficiency and frame error rate.

In this manner, we obtained two sets of $m = 10^9$ (complex) symbols over the same band that was occupied by the quantum data signal shown in the inset of Supplementary Figure 2(left). Denoting these two sets by $Y^{(\mathrm{vac})}$ and $Y^{(\mathrm{elec})}$, the shot noise variance is estimated by

$$\hat{V}_{\mathrm{shot}} = \mathrm{Var}\left(Y^{(\mathrm{vac})}\right) - \mathrm{Var}\left(Y^{(\mathrm{elec})}\right) \ . \tag{67}$$

With ϵ_{cal} as the failure probability for receiver calibration (refer Supplementary Table I), one can assign the upper and lower bound within the confidence interval,

$$V^{+}_{\mathrm{shot}} = (1 + \delta_{\mathrm{Var}}(m, \epsilon_{\mathrm{cal}}/4))\mathrm{Var}\left(Y^{(\mathrm{vac})}\right) - (1 - \delta_{\mathrm{Var}}(m, \epsilon_{\mathrm{cal}}/4))\mathrm{Var}\left(Y^{(\mathrm{elec})}\right) \ , \tag{68}$$

$$V^{-}_{\mathrm{shot}} = (1 - \delta_{\mathrm{Var}}(m, \epsilon_{\mathrm{cal}}/4))\mathrm{Var}\left(Y^{(\mathrm{vac})}\right) - (1 + \delta_{\mathrm{Var}}(m, \epsilon_{\mathrm{cal}}/4))\mathrm{Var}\left(Y^{(\mathrm{elec})}\right) \ . \tag{69}$$

An estimate for the mean photon number of the trusted noise $\hat{t}$ is given by

$$\frac{1}{2} \cdot 2\hat{t} + 1 = \frac{\mathrm{Var}\left(Y^{(\mathrm{vac})}\right)}{\mathrm{Var}\left(Y^{(\mathrm{vac})}\right) - \mathrm{Var}\left(Y^{(\mathrm{elec})}\right)} = \frac{1}{1 - \frac{\mathrm{Var}\left(Y^{(\mathrm{elec})}\right)}{\mathrm{Var}\left(Y^{(\mathrm{vac})}\right)}} \ , \tag{70}$$

where the factor $\frac{1}{2}$ in front of the left hand side of the equation comes from the extra vacuum in heterodyne detection (or equivalently the signal splitting in a phase diverse receiver). Solving for $\hat{t}$ yields

$$\hat{t} = \frac{1}{1 - \frac{\mathrm{Var}\left(Y^{(\mathrm{elec})}\right)}{\mathrm{Var}\left(Y^{(\mathrm{vac})}\right)}} - 1 \ . \tag{71}$$

Since the key fraction is expected to become smaller with less trusted noise (i.e. more untrusted noise) we calculate

$$t = \frac{1}{1 - \frac{1-\delta_{\mathrm{Var}}(m, \epsilon_{\mathrm{cal}}/4)}{1+\delta_{\mathrm{Var}}(m, \epsilon_{\mathrm{cal}}/4)} \frac{\mathrm{Var}\left(Y^{(\mathrm{elec})}\right)}{\mathrm{Var}\left(Y^{(\mathrm{vac})}\right)}} - 1 \ , \tag{72}$$

as the worst-case trusted noise.

SUPPLEMENTARY NOTE 5: RECONCILIATION EFFICIENCY

The information reconciliation (IR) was based on a multi-dimensional (MD) reconciliation scheme and a multi-edge-type low-density-parity-check (MET-LDPC) code of rate 0.190 was used at a SNR of 0.335. The degree distribution

of this code is

$$\nu(\mathbf{r},\mathbf{x}) = 0.1425\,r_1\,x_1^2\,x_2^{13} + 0.0950\,r_1\,x_1^3\,x_2^7 + 0.7625\,r_1\,x_3\;,$$
$$\mu(\mathbf{x}) = 0.0475\,x_1^{12} + 0.5325\,x_2^3\,x_3^1 + 0.230\,x_2^4\,x_3^1\;.$$

Applying this code at a non-optimal estimated SNR of 0.335 obtained from the experimental data, gave us a reconciliation efficiency of $\beta = 91.2$ % while the experimental FER was 2.51×10^{-2} marked by the red pentagon in Supplementary Figure 2(right). In addition, the simulated efficiency versus the average FER for this code is represented by a solid line. For both the simulations and the experimental data, the number of iterations for the LDPC decoder was set to 400.

To achieve a higher reconciliation efficiency with this code rate, we employed a rate-adaptive reconciliation protocol with puncturing [12] to change the rate of the designed MET-LDPC code. The overall MD reconciliation efficiency with puncturing for a dimension dim = 8 is $\beta = R_{\text{punc}}/C_{\text{AWGN}}(s)$, where R_{punc} is the code rate after puncturing. For a codeword of length n and k information bits, the original code rate is $R = k/n$, while after puncturing the final code rate becomes $R_{\text{punc}} = k/(n-p)$, where p denotes the puncturing length. With a puncturing length of 17066, which corresponds to a punctured code rate of $R_{\text{punc}} = 0.197$, the reconciliation efficiency increases to $\beta = 94.3$ % while the obtained FER also rises to 0.121. The corresponding value is shown by the star-shaped marker and the simulation curve is depicted by the dotted line. The main implication of this puncturing is that we require lesser number of symbols to obtain a positive secret key length, however, the final length of the key decreases.

---

[1] A. Leverrier, Physical Review Letters **114**, 10.1103/PhysRevLett.114.070501 (2015), arXiv:arXiv:1408.5689v3.

[2] S. S. Wilks, *Mathematical statistics* (John Wiley, New York, 1962).

[3] C. Forbes, M. Evans, N. Hastings, and B. Peacock, Chi-squared distribution, in *Statistical Distributions* (John Wiley & Sons, 2010) Chap. 11, pp. 69–73, https://onlinelibrary.wiley.com/doi/pdf/10.1002/9780470627242.ch11.

[4] W. H. Press, B. P. Flannery, S. A. Teukolsky, and W. T. Vetterling, *Numerical Recipes in C: The Art of Scientific Computing* (Cambridge University Press, USA, 1988).

[5] S. Pirandola, Physical Review Research **3**, 013279 (2021), arXiv:2010.04168.

[6] T. Gehring, C. Lupo, A. Kordts, D. Solar Nikolic, N. Jain, T. Rydberg, T. B. Pedersen, S. Pirandola, and U. L. Andersen, Nature Communications **12**, 1 (2021).

[7] M. Tomamichel, *A Framework for Non-Asymptotic Quantum Information Theory*, Ph.D. thesis, ETH Zurich (2012), arXiv:1203.2142.

[8] M. Tomamichel, *Quantum Information Processing with Finite Resources – Mathematical Foundations* (Springer Cham, 2015) pp. 1–10, arXiv:1504.00233.

[9] C. Gabriel, C. Wittmann, D. Sych, R. Dong, W. Mauerer, U. L. Andersen, C. Marquardt, and G. Leuchs, Nature Photonics **4**, 711 (2010).

[10] T. Symul, S. M. Assad, and P. K. Lam, Applied Physics Letters **98**, 231103 (2011), https://doi.org/10.1063/1.3597793.

[11] H.-M. Chin, N. Jain, D. Zibar, U. L. Andersen, and T. Gehring, npj Quantum Information **7**, 20 (2021), arXiv:2002.09321.

[12] H. Mani, T. Gehring, P. Grabenweger, B. Ömer, C. Pacher, and U. L. Andersen, Phys. Rev. A **103**, 062419 (2021).
